# Human Papillomavirus 16 E7 Stabilizes APOBEC3A Protein by Inhibiting Cullin 2-Dependent Protein Degradation

**DOI:** 10.1128/JVI.01318-17

**Published:** 2018-03-14

**Authors:** Joseph A. Westrich, Cody J. Warren, Michael J. Klausner, Kejun Guo, Chang-Wei Liu, Mario L. Santiago, Dohun Pyeon

**Affiliations:** a Department of Immunology and Microbiology, University of Colorado School of Medicine, Aurora, Colorado, USA; b Division of Infectious Diseases, Department of Medicine, University of Colorado School of Medicine, Aurora, Colorado, USA; c Department of Biochemistry and Molecular Genetics, University of Colorado School of Medicine, Aurora, Colorado, USA; International Centre for Genetic Engineering and Biotechnology

**Keywords:** APOBEC3, cancer mutagenesis, cervical cancer, cullin, head and neck cancer, innate immunity, papillomavirus, somatic mutation

## Abstract

APOBEC3 (A3) mutation signatures have been observed in a variety of human cancer genomes, including those of cervical and head and neck cancers caused by human papillomavirus (HPV) infection. However, the driving forces that promote off-target A3 activity remain mostly unclear. Here, we report a mechanism for the dramatic increase of A3A protein levels in HPV-positive keratinocytes. We show that expression of the viral protein E7 from high-risk HPVs, but not E7 from low-risk HPVs, significantly prolongs the cellular half-life of A3A protein in human keratinocytes and HPV-positive cancer cell lines. We have mapped several residues within the cullin 2 (CUL2) binding motif of HPV16 E7 as being important for mediating A3A protein stabilization. Furthermore, we provide direct evidence that both A3A and HPV16 E7 interact with CUL2, suggesting that the E7-CUL2 complex formed during HPV infection may regulate A3A protein levels in the cell. Using an *in vitro* cytidine deaminase assay, we show that E7-stabilized A3A remains catalytically active. Taken together, our findings suggest that the HPV oncoprotein E7 dysregulates endogenous A3A protein levels and thus provides novel mechanistic insight into cellular triggers of A3 mutations in HPV-positive cancers.

**IMPORTANCE** Human papillomavirus (HPV) is causally associated with over 5% of all human malignancies. Several recent studies have shown that a subset of cancers, including HPV-positive head and neck and cervical cancers, have distinct mutational signatures potentially caused by members of the APOBEC3 cytidine deaminase family. However, the mechanism that induces APOBEC3 activity in cancer cells is poorly understood. Here, we report that the HPV oncoprotein E7 stabilizes the APOBEC3A (A3A) protein in human keratinocytes by inhibiting ubiquitin-dependent protein degradation in a cullin-dependent manner. Interestingly, the HPV E7-stabilized A3A protein maintains its deaminase activity. These findings provide a new insight into cancer mutagenesis enhanced by virus-induced A3A protein stabilization.

## INTRODUCTION

Human papillomaviruses (HPVs) are small, nonenveloped, double-stranded DNA viruses causally associated with over 5% of all human cancers (1, 2). Persistent infection with high-risk HPV, such as HPV16 and HPV18, is required for HPV-associated cancer progression (3). The HPV oncoprotein E7 plays important roles in cancer progression and maintenance (reviewed in references 4 and 5). HPV E7, lacking inherent enzymatic activity, relies on protein-protein interactions with a myriad of host factors to promote virus replication and persistence (6). Previous studies have shown that high-risk HPV E7 modulates proteasome-mediated protein degradation of several host proteins, including pRB, p107, p130, and PTPN14 (7–11). These proteins are rapidly degraded by ubiquitination through the cullin ubiquitin ligase complex. In contrast, HPV E7 also inhibits degradation of other proteins, such as p53 and p21 (12–14).

The apolipoprotein B mRNA editing enzyme catalytic polypeptide-like 3 (APOBEC3, or A3) family of interferon-inducible cytidine deaminases functions as antiviral restriction factors (reviewed in reference 15). Humans express seven A3 family members: A3A, A3B, A3C, A3D, A3F, A3G, and A3H (16, 17). A3A is notable in that it specifically targets and restricts foreign DNA elements. A3A binds to single-stranded DNA with a high affinity (18), mediates the catabolism of foreign DNA (19), and restricts infection of several DNA viruses, including HPV (20–24). Another study has shown a strong correlation between A3A expression and HPV DNA integration in oropharyngeal cancer, suggesting that HPV episomes in persistent infection may also be targeted by A3A (25).

A3 mutational signatures have been observed in multiple human cancers (26–32). Interestingly, cervical cancer (CxCa) and head and neck cancer (HNC) genomes are enriched with somatic mutations related to A3 cytidine deamination, particularly by A3A and A3B (32–35). We have shown that A3A and A3B mRNA expression is upregulated in HPV-positive keratinocytes and CxCa patient tissues by a mechanism involving the HPV oncoprotein E7 (23). Other studies have also shown that the HPV oncoprotein E6 increases A3B mRNA expression (23, 36–39). These findings imply that cytidine deamination by HPV-induced A3A and/or A3B expression generates somatic mutations in the host genome.

Given that A3A restricts HPV infection (23), HPV must subvert the antiviral activities of A3A to complete its life cycle. To evade A3G restriction, the HIV-1 Vif protein promotes degradation of A3G protein through cullin 5 and the 26S proteasome (40). This process is strikingly similar to HPV E7-mediated pRB degradation mediated by cullin 2 (CUL2) (7). Therefore, we initially hypothesized that HPV counters A3A by promoting its degradation via an E7-dependent process. Contrary to our hypothesis, we found that A3A protein levels were highly elevated in HPV-positive keratinocytes and cancer cell lines. We further revealed that high-risk HPV E7s inhibit the natural turnover of A3A protein by interfering with cullin-mediated degradation of A3A. Our findings suggest that A3A protein stabilized by HPV E7 may contribute to the accumulation of A3 mutational signatures in host cells persistently infected with HPV.

## RESULTS

### HPV16 E7 increases A3A protein levels.

**FIG 1:**
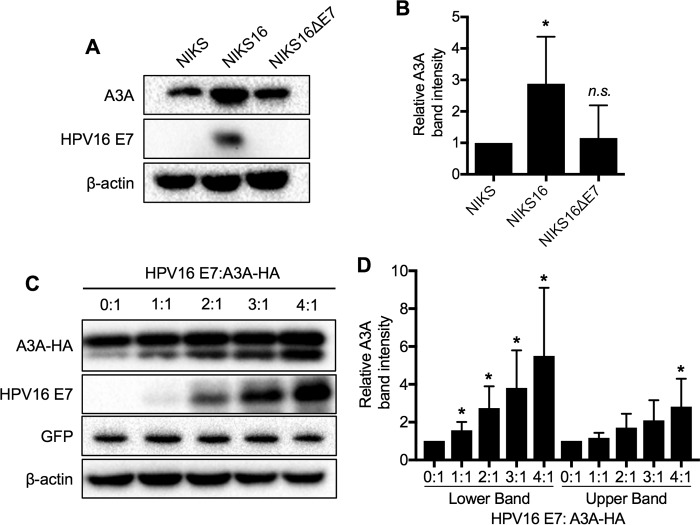
HPV16 E7 increases A3A protein levels. Protein levels of endogenous A3A in NIKS, NIKS-16, and NIKS-16ΔE7 cells (A and B) and exogenous A3A in 293FT cells (C and D) were determined by Western blotting and densitometry. (C) 293FT cells were cotransfected with a fixed amount of pcDNA3.1-A3A-HA and pEGFP-N3 and increasing amounts of pCMV-16E7. (A and C) Cell lysates were prepared, and A3A (endogenous A3A or transfected A3A-HA), HPV16 E7, and β-actin were detected by Western blotting. (C) Transfection efficiency was determined by Western blotting of GFP. (B and D) The A3A band density was normalized to the β-actin band density. Data are shown as the fold changes of normalized A3A band density ± standard deviations relative to the results for NIKS cells (B) or 293FT cells without E7 expression (D). Shown are representative results of four independent experiments. *P* values were calculated by the Student *t* test. *, *P* < 0.05; n.s., not significant.

Although A3A mRNA expression is upregulated in HPV-positive patient tissue samples and cultured keratinocytes (23), A3A mRNA levels may not precisely reflect A3A protein levels. Thus, we examined endogenous A3A protein levels in normal immortalized keratinocytes (NIKS cells), NIKS cells stably harboring episomal HPV16 genomes (NIKS-16), and NIKS-16 cells lacking E7 expression (NIKS-16ΔE7). Our results demonstrated that A3A protein levels were increased by ∼3-fold in the NIKS-16 cells compared to the NIKS cells (Fig. 1A and B). In contrast, the A3A protein level in NIKS-16ΔE7 cells was not significantly different from that in NIKS cells (Fig. 1A and B). This result is consistent with the A3A mRNA levels in NIKS, NIKS-16, and NIKS-16ΔE7 cells shown in our previous study (23). Next, to determine if HPV16 E7 expression is sufficient to increase A3A protein levels, 293FT cells were cotransfected with a constant amount of a hemagglutinin (HA)-tagged A3A expression plasmid (A3A-HA) and increasing concentrations of an HPV16 E7 expression plasmid. Transfection efficiency was determined by cotransfection of a fixed amount of a green fluorescent protein (GFP) expression plasmid. After 72 h, the A3A-HA protein was analyzed by Western blotting. As previously shown by ourselves and others, transfected A3A, unlike endogenous A3A, shows two distinct bands by alternative initiation from a second start site found 36 bases after the canonical start site (Fig. 1C) (41, 42). Our results showed that A3A protein levels were increased by HPV16 E7 expression in a dose-dependent manner (Fig. 1C and D). These results suggest that HPV16 E7 expression is sufficient to increase A3A protein levels.

### HPV16 E7 prevents A3A protein degradation.

**FIG 2:**
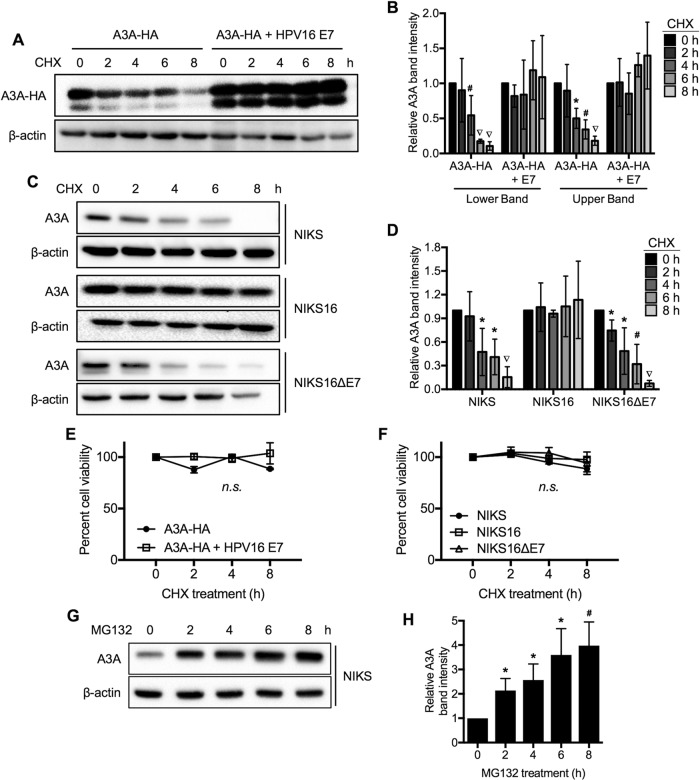
HPV16 prevents A3A protein degradation. (A and B) 293FT cells were cotransfected with pcDNA3.1-A3A-HA and pCMV-16E7 or a corresponding vector. Cotransfected 293FT cells (A, B, and E) and NIKS, NIKS-16 or NIKS-16ΔE7 cells (C, D, and F) were treated with 50 μg/ml cycloheximide (CHX) for the indicated times. (G and H) NIKS cells were treated with 20 μM MG132 for the indicated times. A3A protein expression was analyzed as described in the legend to Fig. 1. Transfected A3A-HA (A) or endogenous A3A (C and G) was detected by Western blotting using anti-HA or anti-A3A antibodies, respectively, and quantified by densitometry as described in the legend to Fig. 1 (B, D and H). The viability of CHX-treated 293FT cells (E) or NIKS, NIKS-16, and NIKS-16ΔE7 cells (F) was assessed using the CellTiter-Glo luminescent cell viability assay (Promega). Data are shown as percent cell viability ± standard deviation and normalized to the viability of untreated (0 h) cells (E and F). All experiments were repeated three times. Data are shown as the fold change relative to the results for cells at 0 h of treatment ± standard deviations. *P* values were determined by the Student *t* test (B, D, and H) or one-way ANOVA (E and F). *, *P* < 0.05; #, *P* < 0.005; ▽, *P* < 0.0005; n.s., not significant.

To determine if HPV16 E7 modulates A3A protein stability, we assessed the natural turnover of A3A protein in 293FT cells cotransfected with A3A-HA and HPV16 E7. Cycloheximide (CHX), which prevents *de novo* protein synthesis, was used to assess the posttranslational stability of A3A protein. Cotransfected 293FT cells were harvested at 0, 2, 4, 6, and 8 h after CHX treatment, and A3A-HA protein was detected by Western blotting. In the absence of HPV16 E7 expression, the majority of A3A protein was degraded within the 8-h time course (Fig. 2A and B). In contrast, HPV16 E7 expression dramatically stabilized both the large and small isoforms of A3A-HA. To determine if HPV16 E7 similarly protects endogenous A3A protein from degradation, NIKS, NIKS-16, and NIKS-16ΔE7 cells were treated with CHX and A3A protein levels were determined. Consistent with our results from exogenous expression of A3A, NIKS-16 cells showed minimal degradation of A3A protein up to 8 h after CHX treatment, while A3A protein in both NIKS and NIKS-16ΔE7 cells was degraded over the time course (Fig. 2C and D). We tested cell viability and found no significant effect of CHX treatment on the viability of NIKS cells (Fig. 2E and F). Next, to test if A3A is degraded by a proteasome-dependent mechanism, we treated NIKS cells with the proteasome inhibitor MG132 and examined A3A protein levels over a time course. We found that blocking proteasome function results in the rapid accumulation of A3A protein in NIKS cells (Fig. 2G and H), indicating that proteasome-dependent protein degradation plays a key role in the natural turnover of A3A protein. Taken together, our results suggest that HPV16 E7 expression stabilizes A3A protein levels in human keratinocytes by preventing proteasome-dependent A3A protein degradation.

### High-risk HPV E7, but not low-risk HPV E7, prevents A3A protein degradation.

**FIG 3:**
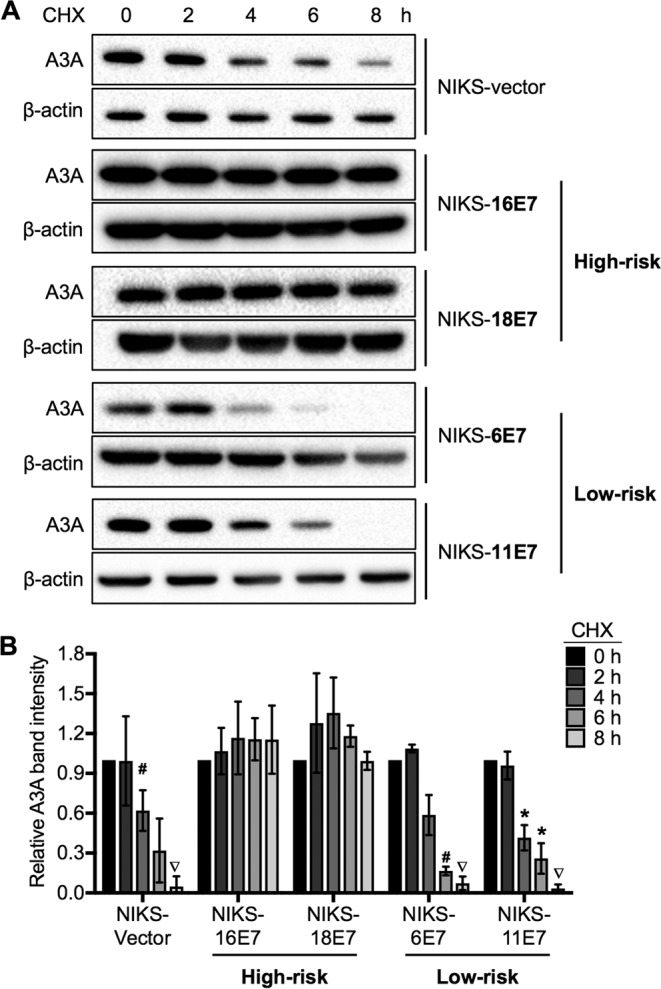
High-risk HPV E7s, but not low-risk HPV E7s, prevent A3A protein degradation. (A) NIKS cells stably expressing HPV6 (NIKS-6E7), HPV11 (NIKS-11E7), HPV16 (NIKS-16E7), or HPV18 (NIKS-18E7) or containing vector alone (NIKS-vector) were treated with 50 μg/ml CHX and analyzed by Western blotting using anti-A3A and anti-β-actin antibodies as described in the legend to Fig. 1. (B) The A3A band density was normalized to the β-actin band density. *P* values were determined by the Student *t* test. *, *P* < 0.05; #, *P* < 0.005; ▽, *P* < 0.0005.

Previous studies have shown that high- and low-risk HPV E7 proteins differentially regulate the degradation of host proteins, most notably, pRB (43, 44). Thus, we hypothesized that E7s from high-risk HPV genotypes, such as HPV16 and HPV18, stabilize A3A protein more efficiently than E7s from low-risk HPV genotypes, such as HPV6 and HPV11. To test this hypothesis, we utilized NIKS cell lines engineered to stably express the E7s of HPV6, HPV11, HPV16, or HPV18 (NIKS-6E7, NIKS-11E7, NIKS-16E7, and NIKS-18E7 cells, respectively) (45). These cells were treated with CHX for 0, 2, 4, 6, and 8 h, and endogenous A3A protein levels were analyzed by Western blotting. Consistent with the results shown for NIKS-16 cells (Fig. 2C), both NIKS-16E7 and NIKS-18E7 cells showed no detectable degradation of A3A protein up to 8 h after CHX treatment (Fig. 3A and B). This result indicates that expression of high-risk HPV E7 is sufficient for preventing endogenous A3A protein degradation in human keratinocytes. In contrast, NIKS-6E7 and NIKS-11E7 cells expressing low-risk HPV E7 showed rapid degradation of A3A protein similar to NIKS and NIKS-vector cells (Fig. 3A and B). These results suggest that high-risk HPV E7s, but not low-risk HPV E7s, prevent A3A protein degradation in human keratinocytes.

### A3A protein is stabilized in HPV-positive CxCa and HNC cell lines.

**FIG 4:**
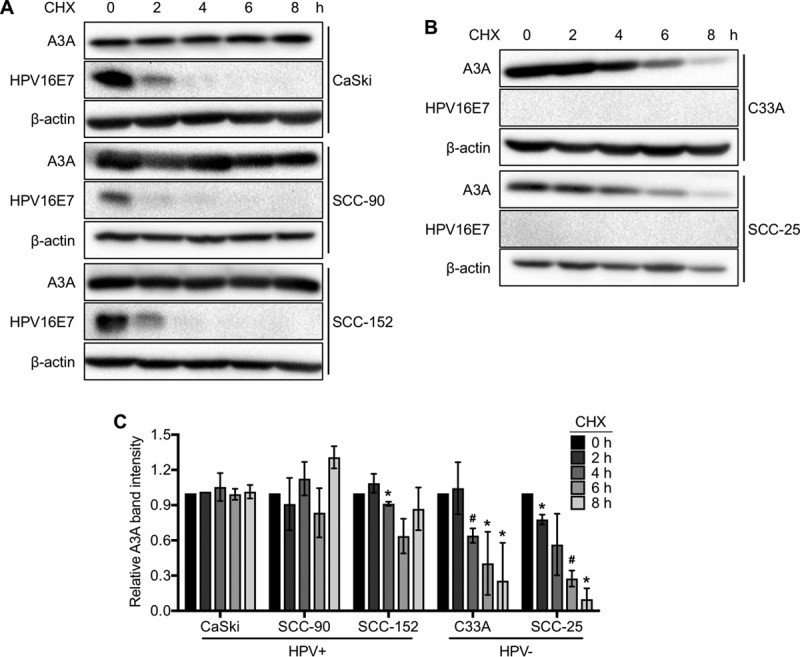
A3A protein is stabilized in HPV-positive CxCa and HNC cell lines. (A and B) HPV-positive CxCa (CaSki) and HNC (SCC-90 and SCC-152) cell lines (A) and HPV-negative CxCa (C33A) and HNC (SCC-25) cell lines (B) were treated with 50 μg/ml CHX for the indicated times and analyzed by Western blotting using anti-A3A, anti-HPV16 E7, and anti-β-actin antibodies as described in the legend to Fig. 1. (C) The A3A band density was normalized to the β-actin band density. Data are shown as the fold change from the results for cells at 0 h of treatment ± standard deviations. Shown are representative results of at least two independent experiments. *P* values were calculated by the Student *t* test. *, *P* < 0.05; #, *P* < 0.005.

To determine if stabilization of A3A protein also occurs in HPV-positive cancer cells, we evaluated the turnover of A3A protein in HPV-positive CxCa (CaSki) and HNC (SCC-90 and SCC-152) cell lines and compared it to that in HPV-negative CxCa (C33A) and HNC (SCC-25) cell lines. The results showed that A3A protein was minimally degraded for 8 h after CHX treatment in all HPV-positive cancer cells (CaSki, SCC-90, and SCC-152) (Fig. 4A and C). In contrast, A3A protein was gradually degraded over the time course in both HPV-negative CxCa (C33A) and HNC (SCC-25) cells (Fig. 4B and C). These results suggest that A3A protein is stabilized in cancer cells by HPV, consistent with our observation in normal immortalized keratinocytes. Notably, we found that HPV16 E7 protein in the HPV-positive cells is rapidly degraded within 2 h (Fig. 4A). These results suggest that although A3A stabilization requires high-risk HPV E7, stability of A3A is maintained after an initial action of HPV E7.

### The CUL2 binding site in HPV16 E7 is important for A3A protein stabilization.

**FIG 5:**
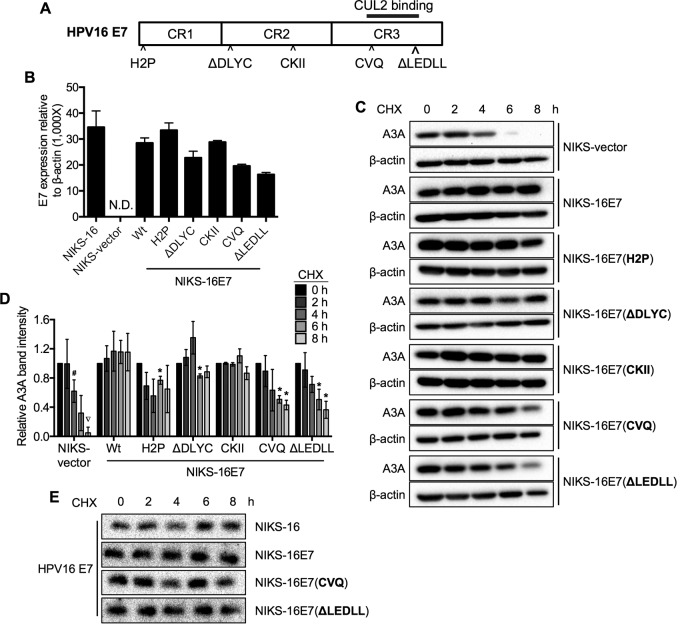
The CUL2 binding site in HPV16 E7 is important for A3A protein stabilization. (A) Schematic diagram of HPV16 E7 protein indicating mutations (^), conserved regions (CR1-3), and CUL2 binding domain (solid black bar). (B) NIKS cells stably expressing wild-type or mutant HPV16 E7 (H2P, ΔDLYC, CKII, CVQ, and ΔLEDLL) or containing the vector alone were generated by lentiviral transduction and 3 μg/ml puromycin selection. Total RNA was extracted from established NIKS cells, and the mRNA expression levels of wild-type and mutant HPV16 E7s were measured by RT-qPCR using specific primers and normalized to the β-actin mRNA levels. Data are shown as the HPV16 E7 mRNA copy number relative to the β-actin mRNA copy number ± standard deviations. N.D., not detected. (C, D) Established NIKS cells were treated with 50 μg/ml CHX, and A3A protein levels were analyzed as described in the legend to Fig. 1. (E) HPV16 E7 protein levels from CHX-treated NIKS-16, NIKS-16E7, NIKS-16E7(CVQ), and NIKS-16E7(ΔLEDLL) cells were analyzed as described in the legend to Fig. 1. Data are shown as the fold changes from the results for cells at 0 h of treatment ± standard deviations. Shown are representative results of two independent experiments. *P* values were determined by the Student *t* test. *, *P* < 0.05; #, *P* < 0.005; ▽, *P* < 0.0005. Wt, wild type.

To determine the mechanism by which high-risk HPV E7 stabilizes A3A protein, we tested five well-characterized HPV16 E7 mutants: H2P, ΔD21-24C (ΔDLYC), SS31-32AA (CKII), CVQ68-70AAA (CVQ), and ΔL79-L83 (ΔLEDLL) (46). These HPV16 E7 mutants result in the loss of pRB degradation (H2P), the inability to bind pRB (ΔDLYC), a lack of phosphorylation by casein kinase II (CKII), and failure to interact with host proteins, including histone deacetylases (HDAC), p21, and CUL2 (CVQ and ΔLEDLL) (Fig. 5A) (reviewed in reference 6). Stable NIKS cell lines expressing each of these HPV16 E7 mutants were established by lentiviral transduction and puromycin selection. The expression of HPV16 E7 mutants was validated by reverse transcriptase quantitative PCR (RT-qPCR) (Fig. 5B). NIKS cells stably expressing E7 mutants or containing vector alone were treated with CHX, and endogenous A3A protein levels were analyzed by Western blotting. Interestingly, NIKS-16E7(CVQ) and NIKS-16E7(ΔLEDLL) cells showed the gradual degradation of A3A protein over the time course, while NIKS cells expressing the other HPV16 E7 mutants consistently stabilized A3A protein, similar to the findings for cells expressing wild-type HPV16 E7 (Fig. 5C and D). Unlike the NIKS-vector cells, A3A protein was not completely degraded in either NIKS-16E7(CVQ) or NIKS-16E7(ΔLEDLL) cells at 8 h after CHX treatment. This suggests that the mutations in each of these two E7 domains are not sufficient to completely block the E7-mediated A3A protein stabilization. To determine if the reduction of A3A stabilization observed for the CVQ and ΔLEDLL mutants was due to expression differences, E7 protein levels were determined in CHX-treated NIKS-16, NIKS-16E7, NIKS-16E7(CVQ), and NIKS-16E7(ΔLEDLL) cells. We noted that the various E7 proteins were stable throughout the time course in each of the groups (Fig. 5E), unlike in HPV-positive CxCa and HNC cell lines (Fig. 4A). However, the initial levels of E7 protein were comparable between wild-type and mutant E7, and the decreased A3A stability in NIKS-16E7(CVQ) and NIKS-16E7(ΔLEDLL) cells was not caused by different E7 expression levels. The regions spanning the CVQ and LEDLL domains in HPV16 E7 are responsible for E7 interaction with CUL2, which is a core component of cullin-RING-based E3 ubiquitin ligase complexes involved in proteasome-mediated pRB degradation (7, 47). Since low-risk HPV E7 does not bind to CUL2 (7), these results are in accordance with the differential functions in A3A protein stabilization between high-risk and low-risk HPV E7s shown in Fig. 3. Thus, our findings imply that A3A protein stabilization by high-risk HPV E7 is, at least in part, mediated by the E7 interaction with CUL2 in the ubiquitin ligase complex.

### CUL2 is necessary for A3A degradation.

**FIG 6:**
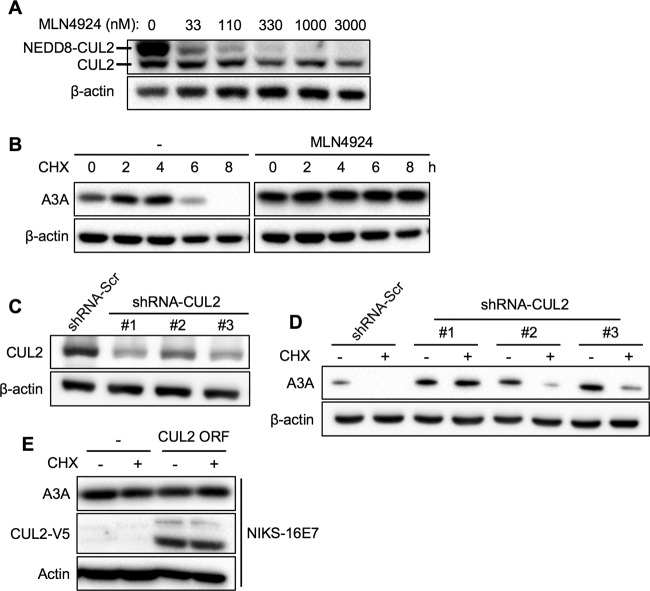
A3A degradation requires CUL2 expression and neddylation. (A) NIKS cells were treated with MLN4924 at the indicated concentrations or vehicle for 8 h. Neddylated CUL2 (NEDD8-CUL2) and CUL2 proteins were analyzed by Western blotting using anti-CUL2 antibody. (B) NIKS cells were treated with 50 μg/ml CHX and either vehicle (dimethyl sulfoxide) or 330 nM MLN4924 for 0, 2, 4, 6, and 8 h. Endogenous A3A and β-actin proteins were analyzed by Western blotting using anti-A3A and anti-β-actin antibodies, respectively. (C) NIKS cells stably expressing scrambled shRNA (shRNA-Scr) or three clones of CUL2-specific shRNA (shRNA-CUL2 #1 to shRNA-CUL2 #3) were generated by lentiviral transduction and 3-μg/ml puromycin selection. CUL2 and β-actin protein expression was detected using anti-CUL2 and anti-β-actin antibodies, respectively. (D) Established NIKS cells were treated with 50 μg/ml CHX, and A3A and β-actin proteins were analyzed by Western blotting as described in the legend to Fig. 1. (E) NIKS-16 cells stably expressing CUL2 were generated by lentiviral transduction of the CUL2-V5 open reading frame (ORF), followed by 10 μg/ml blasticidin selection. Established NIKS-16 cells were treated with 50 μg/ml CHX for 8 h, and CUL2, A3A, and β-actin proteins were analyzed by Western blotting using anti-V5, anti-A3A, and anti-β-actin antibodies, respectively. Shown are representative results of two independent experiments.

Previous studies have shown that activation of cullin activity requires neddylation, which is a posttranslational modification that covalently conjugates a ubiquitin-like protein, NEDD8 (neural precursor cell expressed, developmentally downregulated 8) (48). Treatment of NIKS cells with the neddylation inhibitor MLN4924 led to a dose-dependent reduction in NEDD8-conjugated CUL2 (Fig. 6A). To determine if inhibition of cullin neddylation abrogates A3A protein degradation, CHX-treated NIKS cells were incubated with MLN4924 for 0 to 8 h and A3A protein levels were assessed. Interestingly, MLN4924 treatment prevented A3A protein degradation, similar to the findings for high-risk HPV E7 expression (Fig. 6B). To further determine if CUL2 is required for A3A degradation, we knocked down CUL2 expression in NIKS cells using lentiviral transduction of three clones of short hairpin RNA (shRNA) against CUL2 (shRNA-CUL2 #1 to shRNA-CUL2 #3). We confirmed that CUL2 expression in NIKS cells was significantly decreased by shRNA-CUL2 compared to the scrambled shRNA (shRNA-Scr) (Fig. 6C). Next, to determine if CUL2 knockdown abrogates A3A degradation, we treated the NIKS cells expressing shRNA-CUL2 or shRNA-Scr with CHX for 8 h and measured A3A protein levels. Interestingly, A3A protein degradation was inhibited by CUL2 knockdown in NIKS cells (Fig. 6D). Particularly, shRNA-CUL2 #1 and shRNA-CUL2 #3, which knocked down CUL2 expression more efficiently than shRNA-CUL2 #2, showed significant inhibition of A3A protein degradation in NIKS cells (Fig. 6C and D). Since high-risk HPV E7 degrades other host proteins through CUL2-dependent mechanisms, it is possible that A3A stabilization is a consequence of the limited CUL2 protein otherwise occupied by E7. To determine if A3A stabilization is due to a shortage of CUL2, we overexpressed CUL2 protein in NIKS-16 cells and measured A3A protein levels. The result showed that CUL2 overexpression in NIKS-16 cells did not affect A3A protein stabilization by HPV16 E7 (Fig. 6E). Taken together, our results suggest that CUL2 plays an important role in A3A protein degradation, which is inhibited by high-risk HPV E7.

### CUL2 interacts with A3A and HPV16 E7.

**FIG 7:**
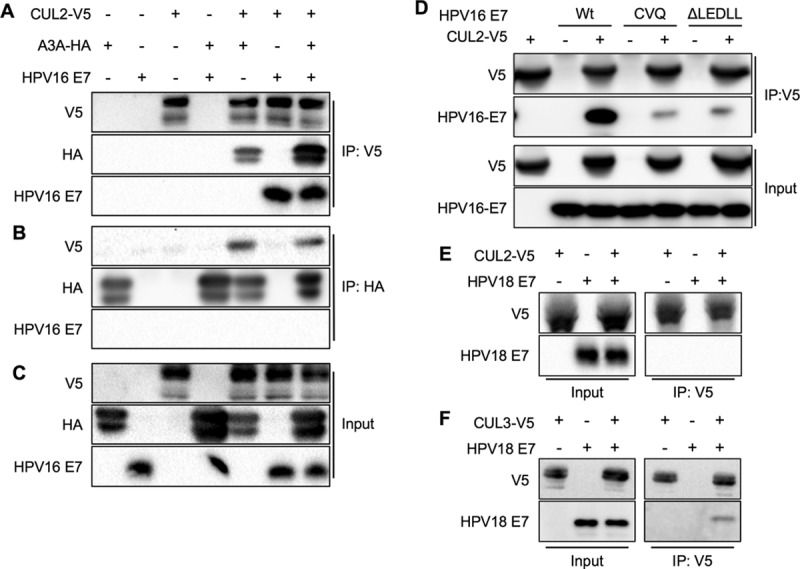
CUL2 interacts with A3A and HPV16 E7 proteins. 293FT cells were cotransfected with expression plasmids of A3A-HA (A to C), CUL2-V5 (A to E), CUL3-V5 (F), HPV16 E7 (A to D), HPV16 E7(CVQ) (D), HPV16 E7(ΔLEDLL) (D), and/or HPV18 E7 (E and F). At 72 h posttransfection, cells were lysed and CUL-V5 and A3A-HA proteins were pulled down using anti-V5 (A, D to F) and anti-HA (B) antibodies, respectively. Each protein in the input and pulldown samples was detected by Western blotting using anti-V5, anti-HA, anti-HPV16 E7, and anti-HPV18 E7, as indicated. Shown are representative results of at least two independent experiments. IP, immunoprecipitation.

HPV16 E7 directly interacts with CUL2 for pRB degradation (7). To determine if CUL2 interacts with A3A in the presence or absence of HPV16 E7, 293FT cells were cotransfected with V5-tagged CUL2, A3A-HA, and/or HPV16 E7. At 72 h posttransfection, cells were harvested and the CUL2 and A3A proteins were pulled down using anti-V5 and anti-HA antibodies, respectively. The results showed that CUL2 was coimmunoprecipitated with both A3A and HPV16 E7 (Fig. 7A). However, A3A interacted only with CUL2 and not directly with HPV16 E7 (Fig. 7B). The input A3A-HA, CUL2-V5, and HPV16 E7 proteins were confirmed by Western blotting (Fig. 7C). These results suggest that both A3A and HPV16 E7 interact directly with CUL2 and that A3A protein stabilization by HPV16 E7 is not mediated through a direct protein interaction between A3A and HPV16 E7. Given that the HPV16 E7 mutants CVQ and ΔLEDLL are less effective for A3A protein stabilization (Fig. 5C and D), we next determined whether CUL2 interacts with these mutants. Interestingly, our results revealed that CUL2 interaction with HPV16 E7 with the CVQ and ΔLEDLL mutations was significantly reduced compared to that with wild-type HPV16 E7 (Fig. 7D). This suggests that the reduced A3A stabilization by the CVQ and ΔLEDLL mutations was due to the poor ability of the mutants with these mutations to interact with CUL2.

HPV18 E7 is also capable of stabilizing A3A protein (Fig. 3A). However, a previous study showed that HPV18 E7 does not interact with CUL2 (7), and our data are consistent with this observation (Fig. 7E). We next tested whether HPV18 E7 interacts with another candidate cullin family member, CUL3, using cotransfection of CUL3-V5 and HPV18 E7. Interestingly, we found that HPV18 E7 directly interacts with CUL3, although the binding capacity was modest (Fig. 7F). This result suggests that CUL3, instead of CUL2, may contribute to A3A stabilization by HPV18 E7. Taken together, CUL2 appears to be integral in the stabilization of A3A by HPV16 E7 and additional cullins may be involved in A3A stabilization by other high-risk HPV genotypes.

### HPV16 E7-stabilized A3A maintains deaminase activity.

**FIG 8:**
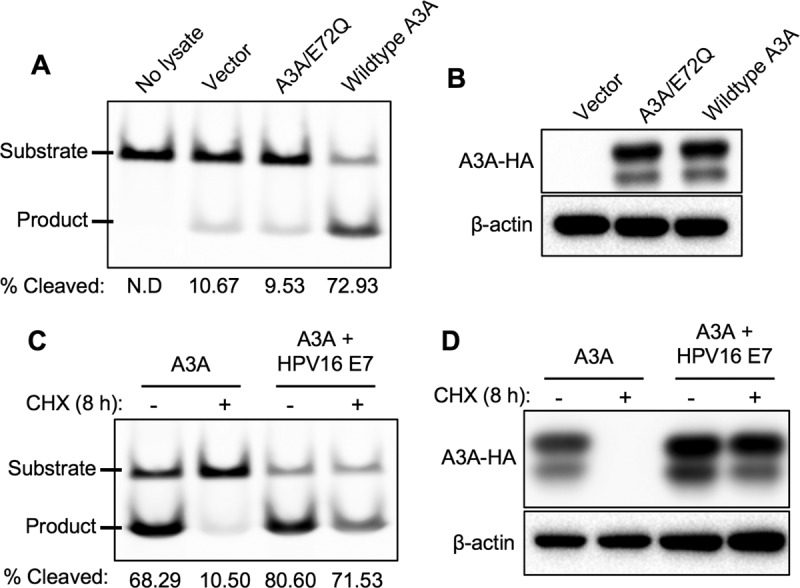
HPV16 E7-stabilized A3A maintains deaminase activity. (A and B) 293FT cells were transfected with vector only, wild-type A3A-HA, or A3A/E72Q-HA for 72 h. Phosphate-buffered saline was used for the no-lysate negative control. (B) Wild-type and mutant A3A expression was validated by Western blotting. (C) 293FT cells were transfected with A3A-HA alone or both A3A-HA and HPV16 E7 for 72 h and treated with vehicle or 50 μg/ml CHX as described in the legend to Fig. 1. (A and C) Cell lysates were prepared, and an *in vitro* cytidine deaminase assay was performed using TC-containing oligonucleotides as described in Materials and Methods. The reaction products were analyzed on 15% polyacrylamide-urea gel, and percent cleavage was determined by densitometry using the following formula: percent cleavage = [amount of product/(amount of substrate + amount of product)] × 100. (D) A3A expression was validated by Western blotting. Shown are representative results of two independent experiments.

To determine if the A3A protein stabilized by HPV16 E7 retains its cytidine deaminase activity, an *in vitro* DNA deamination assay was performed using a fluorescently tagged oligonucleotide containing a single A3 target motif (TC) (36, 49). First, to validate the cytidine deaminase assay, cell lysates were prepared from 293FT cells transiently transfected with wild-type A3A, a catalytically inactive A3A mutant (A3A/E72Q), or the vector only. While the vector only and A3A/E72Q expression showed minimal cleavage of TC-containing oligonucleotides, wild-type A3A expression resulted in a significant increase of cleavage (Fig. 8A). Expression of wild-type and mutant A3A proteins in transfected cell lysates was validated by Western blotting (Fig. 8B). To determine the deaminase activity of HPV16 E7-stabilized A3A protein, 293FT cells were transfected with A3A alone or both A3A and HPV16 E7, followed by CHX treatment. In cell lysates where A3A protein was stabilized by HPV16 E7, we observed deaminase activity dramatically higher than that in control cell lysates (Fig. 8C and D). These results suggest that A3A protein stabilized by high-risk HPV E7 is functionally active and is capable of deaminating target cytosine residues.

### A3A protein stabilization by HPV16 E7 is independent of TRIB3.

**FIG 9:**
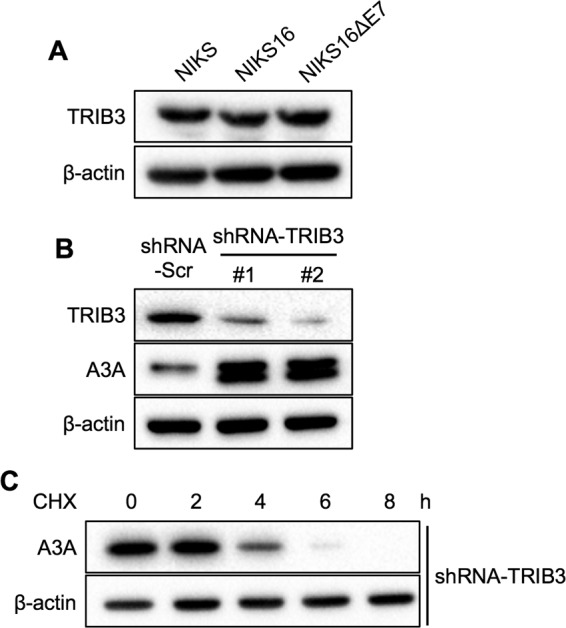
A3A protein stabilization by high-risk HPV E7 is independent of TRIB3. (A) Endogenous TRIB3 protein in NIKS, NIKS-16, and NIKS-16ΔE7 cells was detected by Western blotting using anti-TRIB3 antibody. NIKS cells stably expressing scrambled shRNA (shRNA-Scr) or two TRIB3-specific shRNA clones (shRNA-TRIB3 #1 and shRNA-TRIB3 #2) were generated by lentiviral transduction and 3 μg/ml puromycin selection. (B) TRIB3, A3A, and β-actin proteins were detected using anti-TRIB3, anti-A3A, and anti-β-actin antibodies, respectively. (C) NIKS cells with TRIB3 knockdown were treated with 50 μg/ml CHX, and A3A and β-actin proteins were analyzed by Western blotting.

Tribbles homolog 3 (TRIB3) is a pseudokinase that inhibits the AKT/mTOR (mammalian target of rapamycin) pathway (50). A previous study showed that TRIB3 facilitates A3A protein degradation (51). To test whether A3A protein stabilization is caused by HPV16 E7 inhibition of TRIB3 functions, we first determined endogenous TRIB3 protein levels in NIKS, NIKS-16, and NIKS-16ΔE7 cells. Our result showed no detectable difference in the expression levels of TRIB3 protein in NIKS-16 cells compared to NIKS and NIKS-16ΔE7 cells (Fig. 9A).

To determine the effect of TRIB3 on A3A protein levels in human keratinocytes, we knocked down TRIB3 expression in NIKS cells by lentiviral transduction of shRNA against TRIB3 (shRNA-TRIB3). Consistent with the previous finding (51), endogenous A3A protein levels were increased in NIKS cells by TRIB3 knockdown (Fig. 9B). Next, to determine if TRIB3 knockdown prevents A3A protein turnover in NIKS cells, NIKS cells containing shRNA-TRIB3 #2 were treated with CHX and A3A protein levels were analyzed by Western blotting. The results showed that TRIB3 knockdown did not affect A3A protein degradation in NIKS cells (Fig. 9C). These results suggest that A3A protein stabilization by high-risk HPV E7 is independent of TRIB3.

## DISCUSSION

HPVs are causally associated with multiple human cancers, including CxCa and a subset of HNC (52, 53). While the tumor suppressors p53 and pRB are rapidly inactivated by expression of the HPV oncoproteins E6 and E7, respectively, HPV-associated carcinogenesis requires decades of disease progression to develop invasive cancer (3). During this process, HPV persists in host cells and continuously contributes to cancer progression (54, 55). The underlying mechanisms of this slow process are largely unknown.

The majority of cancers are driven by somatic mutations that accumulate over decades. Several mechanisms of somatic mutagenesis in cancer are well understood. For example, smoking and UV light exposure induce mutations that contribute to lung and skin cancer development, respectively (29). In contrast, the drivers of somatic mutations for other cancers, including HPV-positive HNC and CxCa, were completely unknown until only recently. Several recent studies have shown that A3-associated mutation signatures are highly enriched in multiple human cancers, including HNC and CxCa (28, 29, 32, 56). Our previous study revealed that A3A and A3B mRNA expression is upregulated in HPV-positive keratinocytes in an HPV oncoprotein-dependent manner (23). Interestingly, A3A, but not A3B, potently restricts HPV infection. A recent study by Chan and colleagues showed that A3A is likely the predominant mutagenic A3 in cancers, causing over 10-fold higher A3-specific mutations than A3B (57). Nevertheless, the increase of A3A mRNA expression in HPV-positive HNC and CxCa is modest compared to the increase of A3B mRNA expression levels (23, 36). This implies that there may be other mechanisms to enhance off-target A3A activity in HPV-positive cells.

Here, we report that A3A protein accumulates in HPV-positive cells through HPV E7-mediated protein stabilization. HPV E7 modulates protein degradation and stabilization through mechanisms dependent on or independent of the ubiquitin ligase complex (12, 13, 58–60). While it is well-known that the ubiquitin ligase complex is involved in promoting protein degradation, other studies have shown that the ubiquitin ligase complex also plays important roles in protein stabilization (61–63).

High-risk HPV E7 binds to pRB with a high affinity compared to low-risk HPV E7 and degrades pRB through the CUL2-based E3 ubiquitin ligase complex (7, 64, 65). Cullins are members of the ubiquitin ligase complex that play critical roles in mediating the degradation of a myriad of cellular proteins (7, 65). Interestingly, our results showed that high-risk HPV E7s, but not low-risk HPV E7s, inhibit proteasome-dependent degradation of A3A. Further, HPV16 E7 mutants (CVQ and ΔLEDLL) defective for interaction with CUL2 did not completely stabilize A3A protein. This diminished capacity to stabilize A3A may be due to a restricted interaction between HPV16 E7 and CUL2 by the CVQ and ΔLEDLL mutations. These results suggest that CUL2 plays an important role in A3A protein stabilization by HPV16 E7.

Viral proteins frequently target ubiquitin ligase components to modulate protein degradation. Merkel cell polyomavirus small T antigen stabilizes viral large T antigen by inhibiting the cellular SCF(Fbw7) E3 ligase (66). To induce lymphoproliferation, murine gammaherpesvirus stabilizes host Myc protein through the viral E3 ubiquitin ligase mLANA (67). Hepatitis B virus X protein also inhibits Myc protein degradation by targeting the SCF(Skp2) ubiquitin E3 ligase (68). Previous studies have shown that HPV16 E7 stabilizes several host proteins, including HIF-1α, p53, and p21 (12, 13, 69). Interestingly, all these proteins stabilized by HPV16 E7 are naturally degraded through the CUL2-based E3 ubiquitin ligase complex (70). Degradation of pRB and HIF-1α proteins requires neddylation of CUL2 (48, 65). Our results also show that inhibition of neddylation stabilizes A3A protein, suggesting that cullin neddylation is required for A3A protein degradation. Further, knockdown of CUL2 expression inhibits A3A protein degradation, suggesting a pivotal role of CUL2 in A3A degradation. While the interaction of HPV16 E7 with CUL2 has been previously shown (7), no studies have determined if A3A protein interacts with HPV16 E7 and CUL2. Here, we show that CUL2 binds to both HPV16 E7 and A3A individually, but A3A does not directly interact with HPV16 E7. These results suggest that HPV16 E7 likely inhibits A3A protein degradation through a mechanism involving CUL2. However, we found that HPV18 E7 does not interact with CUL2, in agreement with a previous finding (7), indicating that A3A stabilization by HPV18 E7 may be mediated through other cullins. Indeed, we found that HPV18 E7 interacts with CUL3 instead of CUL2. As their interaction is modest compared to the HPV16 E7 and CUL2 interaction, CUL3 alone may not be sufficient to account for the entire mechanism of A3A stabilization. Further investigations are necessary to understand the detailed mechanisms by which different high-risk HPV E7s stabilize A3A protein levels.

HPV E7 has been shown to increase the levels of p53 and p21 proteins while limiting their activity (12–14). In contrast, HPV16 E7 stabilizes A3A while maintaining A3A deaminase activity. The deaminase activity of E7-stabilized A3A may facilitate mutagenesis of the host genome in persistently infected keratinocytes (26, 29, 31). Consistent with our finding of A3A protein stabilization in HPV-positive CxCa and HNC cells (Fig. 4A), a recent study by Kondo et al. has shown highly increased A3A protein levels in HPV-positive HNC tissues compared to HPV-negative HNC tissues (25).

Interestingly, the stability of the E7 protein varies extensively depending on different cell types. HPV16 E7 in HPV-positive CxCa and HNC cells showed a short half-life of less than 2 h, in agreement with previous observations (Fig. 4) (71, 72). In contrast, HPV16 E7 protein was stable for 8 h in NIKS cells (Fig. 5E). The varied stability of E7 has been observed before, as several studies have shown that the half-life of E7 proteins varies depending on different cell lines and the cellular mechanisms involved in protein degradation (73). First, interactions with some cellular proteins, such as HSP90, GRP78, and USP11, significantly increase the steady-state level of E7 (72, 73). Second, another study has shown that E7 protein stability varies depending on its subcellular localization. While nuclear E7 is short-lived, cytosolic E7 forming oligomers is stable over 6 h (74). Third, the half-life of phosphorylated E7 (Ser^33^phospho-E7) is over 24 h (75). Additionally, dual-specificity tyrosine phosphorylation-regulated kinase 1A stabilizes HPV16 E7 protein through phosphorylation of the threonine 5 and threonine 7 residues (76). Therefore, it is possible that E7 overexpressed in NIKS cells may have a long half-life due to one or more of these mechanisms. However, regardless of the differential E7 protein stability, A3A protein was consistently stabilized in HPV-positive cells. As HPV16 E7 does not directly bind to A3A, this suggests that high-risk HPV E7 may initiate early steps of A3A protein stabilization.

Given that A3A restricts HPV infection (23), A3A protein stabilized by HPV E7 could be detrimental to the virus life cycle. Thus, HPV must evade restriction by A3A to successfully establish infection. Strikingly, TC dinucleotides, the preferred target site of A3A, are significantly underrepresented in the genomes of alphapapillomaviruses, which includes all high-risk HPV genotypes (77). Since the basal mRNA expression level of A3A is significantly higher in mucosal skin than cutaneous skin (77), alphapapillomaviruses may have evolved to survive in an environment with high A3A levels. This implies that the HPV genome may be highly resistant to A3A-mediated mutagenesis, while elevated levels of A3A protein by high-risk HPV E7 may contribute to off-target effects on the host. HPV may derive additional benefit from the increased A3A activity. It has been shown that HPV replication relies, in part, on the DNA damage response (DDR) (78–80). Aberrant A3A activity has been shown to activate the DDR response (81) and thus may assist the virus in its replication. Notably, the antiviral effect of A3A appears to be most prominent in the next target cell due to a significant decrease in virion infectivity (23). This phenomenon may be explained by A3A encapsidation into HPV virions, similar to how APOBEC3 proteins inhibit many other viruses. Thus, E7-mediated stabilization of A3A in the producer cell may not necessarily impact HPV replication if it does not affect encapsidation. In fact, E7-CUL2-mediated sequestration may even serve as a viral countermeasure against A3A.

Taken together, our findings imply that high-risk HPV E7 stabilizes A3A protein in persistent HPV infection. This stabilized A3A may contribute to cancer mutagenesis during HPV-associated cancer progression, which generates the A3 mutation signatures observed in HPV-positive HNC and CxCa.

## MATERIALS AND METHODS

### Cell lines and reagents.

293FT cells were purchased from Life Technologies and cultured in Dulbecco's modified Eagle's medium containing 10% fetal bovine serum (FBS). Normal human immortalized keratinocyte (NIKS) cells (obtained from Lynn Allen-Hoffman) (82) and NIKS-16 cells (obtained from Paul Lambert) (83) were maintained with mitomycin C-treated NIH 3T3 cells (obtained from Paul Lambert) in E-medium, as previously described (82). NIKS cell lines expressing various wild-type and mutant E7s (NIKS-6E7, NIKS-11E7, NIKS-16E7, NIKS-18E7, NIKS-16E7 H2P, ΔD21-24C [ΔDLYC], SS31-32AA [CKII], CVQ68-70AAA [CVQ], and ΔL79-L83 [ΔLEDLL]) were established using lentiviral transduction and puromycin (3 μg/ml) selection. NIKS cell and NIKS cell derivatives were maintained under passage 50 and passage 10, respectively, and frequently validated by determination of morphology, HPV early gene expression, and feeder cell dependency. The CaSki (84) and C33A (85) cell lines were obtained from Paul Lambert. The SCC-25, SCC-90, and SCC-152 cell lines were purchased from the American Type Culture Collection (ATCC). All CxCa and HNC cell lines were cultured in Dulbecco's modified Eagle's medium containing 10% FBS. All cell lines were confirmed to be mycoplasma free before use.

### Plasmid constructs.

The hemagglutinin (HA)-tagged A3A (pcDNA3.1-A3A-HA) expression plasmid and its parental vector (pcDNA3.1) were prepared as previously described (86). Wild-type HPV16 E7 (pCMV-16E7) and the HPV16 E7 mutants (the H2P, ΔDLYC, CKII, CVQ, and ΔLEDLL mutants; obtained from Karl Munger) were cloned into a lentiviral plasmid, pCDH-CMV-MCS-EF1-Puro (catalog number CD510B-1; System Bioscience). The green fluorescent protein (GFP) expression plasmid (pEGFP-N3) was obtained from Clontech. High-risk HPV (HPV16 and HPV18) and low-risk HPV (HPV6 and HPV11) E7 expression plasmids were obtained from Joe Mymryk (Western University) and cloned into pCDH-CMV-MCS-EF1-Puro (45). The lentiviral expression plasmids of V5-tagged CUL2 (ccsbBroad304_01930), V5-tagged CUL3 (ccsbBroad304_07243), and V5-tagged TRIB3 (ccsbBroad304_03850) and the shRNA clones against TRIB3 (clones TRCN0000196756 and TRCN0000295919) and CUL2 (clones TRCN0000006522, TRCN0000006523, TRCN0000006525, and TRCN0000006526) were obtained through the Functional Genomics Facility at the University of Colorado School of Medicine.

### Lentivirus production and transduction.

Lentiviruses were produced using 293FT cells transfected with the packaging constructs pMDG.2 (plasmid number 12259; Addgene), psPAX2 (plasmid number 12260; Addgene), and the transfer vector as previously described (23). At 48 and 72 h posttransfection, cell culture supernatants were collected and virus was concentrated using ultracentrifugation at 25,000 × *g* for 3 h. Keratinocytes were spinfected with 100×-concentrated lentiviruses by centrifugation at 1,000 × *g* for 30 min at 37°C, followed by selection using 3 μg/ml puromycin or 10 μg/ml blasticidin.

### Drug treatment and cell viability assays.

To inhibit *de novo* protein synthesis, cells were treated with 50 μg/ml of CHX (CAS number 01810; Sigma). For proteasome inhibition, keratinocytes were treated with 20 μM MG132 (catalog number 133407-82-6; Cayman Chemical). To inhibit cullin neddylation, cells were treated with 33 nM to 3 μM MLN4924 (CAS number 905579-51-3; Cayman Chemical). Cell viability was determined using a CellTiter-Glo luminescent cell viability assay (Promega) according to the supplier's instructions.

### Western blotting.

Cell lysates were prepared and Western blotting was performed as previously described (23). HA-tagged protein was detected using mouse anti-HA antibody (catalog number ab9110; Abcam) at a 1:5,000 dilution. A3A was detected using rabbit anti-A3A antibody (catalog number ab150369; Abcam) at a 1:3,000 dilution. GFP was detected using rabbit anti-GFP (catalog number ab290; Abcam) at a 1:1,000 dilution. CUL2 was detected using rabbit anti-CUL2 antibody (clone number 50H17L12; Thermo Fisher) at a 1:500 dilution. TRIB3 was detected using rabbit anti-TRIB3 antibody (catalog number ab75846; Abcam) at a 1:2,000 dilution. V5-tagged proteins were detected with mouse anti-V5 antibody (catalog number MA5-15253; Thermo Fisher) at a 1:2,000 dilution. Mouse anti-HPV16 E7 (catalog number sc-6981; Santa Cruz Biotechnology) and mouse anti-HPV18 (catalog number sc-365035; Santa Cruz Biotechnology) antibodies were used at a 1:100 dilution. Horseradish peroxidase-conjugated goat anti-rabbit immunoglobulin (catalog number 111-035; Jackson Laboratories) and donkey anti-mouse immunoglobulin (catalog number 715-035 Jackson Laboratories) secondary antibodies were used at a 1:10,000 dilution. Band densities were determined using Bio-Rad Image Lab (version 4.1) software and normalized to the β-actin band intensity.

### Coimmunoprecipitation.

Cell lysates were prepared and incubated with protein A/G agarose beads (Thermo Fisher) at 4°C for 4 h to eliminate nonspecific binding. The lysates were incubated with 1 μg of specific antibody (anti-V5 or anti-HA) or isotype-matched antibody and incubated overnight, followed by incubation with protein A/G agarose beads at 4°C for 15 min. The beads were washed five times, and the bound proteins were analyzed by Western blotting.

### Reverse transcriptase quantitative PCR.

Total RNA was extracted using an RNeasy minikit (Qiagen) with on-column DNase digestion using the RNase-free DNase (Qiagen) according to the supplier's instructions. First-strand cDNA was synthesized using a Transcriptor first-strand cDNA synthesis kit (Roche) from 1 μg of total RNA. Using a Bio-Rad CFT Connect real-time system, real-time PCR was performed in 20 μl of a reaction mixture containing FastStart Universal SYBR green master (carboxy-X-rhodamine [ROX]; Roche Applied Science), 0.5 μM each primer, 10 μl of SYBR green PCR master mix, 5 μl of cDNA template, and nuclease-free water. The primers used in this study included HPV16 E7 set 1 (for the H2P, ΔDLYC, CVQ, and ΔLEDLL E7 mutants) primers 5′-AAATGACAGCTCAGAGGAGGAG-3′ (sense) and 5′-GAGTCACACTTGCAACAAAAGG-3′ (antisense) and HPV16 E7 set 2 (for the CKII E7 mutant) primers 5′-TTTGCAACCAGAGACAACTGAT-3′ (sense) and 5′-GAGTCACACTTGCAACAAAAGG-3′ (antisense). β-Actin was detected using the primers 5′-TCACCCACACTGTGCCCATCTA-3′ (sense) and 5′-TGAGGTAGTCAGTCAGGTCCCG-3′ (antisense).

### Cytidine deaminase assay.

293FT cells were harvested at 72 h posttransfection, and cell lysates were prepared. In each reaction, 5 μl of protein lysate was added to the DNA deaminase reaction mixture containing 5 pmol of 5′ fluorescein-labeled oligonucleotide with a single TC target motif (5′[ATA]_7×_ -TCC-[ATA]_6×_-3′) in deaminase buffer (100 mM Tris HCl, 500 mM NaCl, and 10 mM dithiothreitol). The reaction mixture was incubated at 37°C for 2 h, and then 1 unit of uracil DNA glycosylase was added and the mixture was further incubated at 37°C for 1 h. NaOH (100 nM) was added to the reaction mixture, and the mixture was incubated at 95°C for 10 min. Reaction products were analyzed on a denaturing 15% polyacrylamide-urea gel using a Bio-Rad Molecular Imager FX, as previously described (49, 87, 88).

### Statistical analysis.

Student's *t* test and one-way analysis of variance (ANOVA) were used to calculate significance for comparison of two matched groups or continuous variation between several groups, respectively, using Prism (version 7) software (GraphPad). Results were considered statistically significant at a *P* value of less than 0.05.
